# Age-dependent mitochondrial toxicity in renal stem cells: A precise 3D silk matrix model

**DOI:** 10.1016/j.gendis.2026.102108

**Published:** 2026-02-23

**Authors:** Pengfei Yu, Huifen Ding, Jian-Xing Ma, Zhongping Duan, Yu Chen, Anthony Atala, Yuanyuan Zhang

**Affiliations:** a The Fourth Department of Liver Disease, Beijing YouAn Hospital, Capital Medical University, Beijing 100069, China; b Wake Forest Institute for Regenerative Medicine, Wake Forest University Health Sciences, Winston–Salem, North Carolina 27101, USA; c Department of Biochemistry, Wake Forest University Health Sciences, Winston–Salem, North Carolina 27157, USA

**Keywords:** 3D cell culture, Aging, Drug-induced kidney injury, Mitochondrial function parameters, Renal stem cells, Silk matrix, Tenofovir

## Abstract

Aging is a significant risk factor for drug-induced kidney injury, underscoring the need for advanced preclinical models. The impact of age on the susceptibility of human primary kidney stem cells to drug-induced mitochondrial toxicity remains largely uncharacterized. To address this gap, we employed a well-established *in vitro* 3D model, utilizing a silk fiber matrix, to culture renal stem cells derived from both young and elderly donors. Tenofovir, a commonly prescribed antiretroviral medication linked to renal adverse effects, was used to investigate age-related drug-induced mitochondrial toxicity. Our findings reveal a pronounced age-dependent increase in tenofovir-induced mitochondrial dysfunction in older renal stem cells compared with their younger counterparts. This was characterized by diminished mitochondrial mass and function, ATP production, elevated oxidative stress, senescence gene expression, and compromised renal cell function. These results highlight the critical role of aging in exacerbating drug-induced renal injury. The 3D silk matrix model utilizing elderly donor-derived renal stem cells offers a promising platform for identifying medications with age-specific renal toxicity potential and for developing targeted therapies to safeguard the aging kidney.

## Introduction

Numerous drugs are known to induce renal mitochondrial toxicity (MtT); however, evaluating MtT in human renal cells remains challenging due to the lack of physiologically relevant models. In this study, we employed antiretroviral therapy as a representative agent to investigate age-dependent drug-induced renal MtT using a well-established 3D silk matrix model.

The aging of individuals living with human immunodeficiency virus (HIV) has become a major public health concern, particularly in the context of the rapidly aging population in the United States.^1^ While antiretroviral therapy is highly effective in suppressing HIV infection, it is also associated with significant adverse effects, including renal injury. Globally, the incidence of kidney injury among patients receiving antiretroviral agents is estimated to range from 6.4% to 20%.^2^^,^^3^ Tenofovir (TFV), a nucleoside reverse transcriptase inhibitor, is currently recommended by the WHO as a first-line treatment for HIV and hepatitis B virus (HBV) infections^4^; however, its use has been linked to chronic renal insufficiency, which is believed to result from MtT in proximal renal tubular cells.

Renal stem or progenitor cells, such as urine-derived stem cells (USCs), play a critical role in kidney tissue repair and maintaining renal function. Numerous drugs have been implicated in renal MtT, a key mechanism contributing to kidney injury. TFV, a widely prescribed antiretroviral agent, exemplifies this concern due to its association with renal adverse effects. However, current models lack the ability to specifically assess drug-induced MtT in renal stem cells, and the consequences of such toxicity on these essential cells remain poorly defined. This challenge is further compounded by the natural decline in kidney function with aging, characterized by reduced numbers of renal stem cells and nephrons, as well as progressive vascular stiffening and susceptibility to occlusion. The combination of aging-related changes and TFV-induced MtT may accelerate renal functional decline in older adults,^5^ heightening the risk of chronic renal insufficiency and kidney failure. Nevertheless, the underlying molecular mechanisms remain incompletely understood.

Both tenofovir disoproxil fumarate (TDF) and tenofovir alafenamide (TAF) are prodrugs that are intracellularly converted to TFV, the active agent responsible for MtT in proximal tubular cells. Compared with TDF, TAF exhibits greater plasma stability, resulting in lower systemic TFV exposure and, consequently, a more favorable renal safety profile.^6^^,^^7^ This has been demonstrated in both patients who are new to TFV and in patients who switch from TDF to TAF. However, TAF is a newer drug than TDF, and more long-term data are needed to fully assess its renal safety profile.^8^, ^9^, ^10^ It is important to note that both TDF and TAF can cause chronic renal insufficiency, and the risk is higher in patients with pre-existing kidney damage, such as aging. Therefore, it is critical to investigate age-related changes in telomere dynamics and mitochondrial function in renal stem cells of elderly patients receiving long-term antiretroviral therapy.

Previous studies by our group^11^^,^^12^ and others^13^^,^^14^ have demonstrated the potential of silk matrix as a promising, cost-effective material for 3D renal toxicity models. Its biocompatibility, porosity, and low biodegradability enable the creation of a microenvironment closely resembling native renal tissue, while minimizing immune responses and allowing for controlled degradation. Moreover, the material's tunable mechanical properties, including stiffness and porosity, facilitate the construction of 3D structures that support cell attachment and growth. Leveraging these advantages, we employed the silk matrix platform in this study to develop predictive models for investigating age-related renal toxicity.

The aging-related dysfunction of the telomere mitochondrial axis, crucial in age-related disorders, is the focus of this study investigating TFV-induced telomere mitochondrial dysfunction in USC from healthy older adults (oUSC) compared with those from healthy young adults (yUSC). By exposing an *in vitro* 6-week 3D culture model of human USC to low doses (clinically relevant concentrations) and high doses (10-fold clinically relevant concentrations) of TFV, we assessed telomere parameters and mitochondrial function, including mitochondrial mass, levels of mitochondrial respiratory chain complexes I–V, mitochondrial DNA (mtDNA) content, and renal cell function (kidney injury molecular-1/KIM1 and cytochrome P450 2E1/CYP2E1) of these USCs. We investigated various aspects of MtT, including age-related MtT, TFV-related MtT, and cell sensitivity to drug-related MtT, time-related MtT, and dose-related MtT. Additionally, we investigated the role of peroxisome proliferator-activated receptor γ coactivator 1α (PGC-1α) as a potential mediator of telomere-driven mitochondrial dysfunction in renal progenitor cells from aging kidneys. These findings may inform the development of targeted strategies to prevent or reverse TFV-induced renal damage in vulnerable aging populations by preserving renal stem cell function and integrity. Moreover, this work provides broader insight into age-related chronic renal insufficiency associated with other nephrotoxic agents.

## Materials and methods

### Silk fiber matrix and drugs

Silk fibroin was extracted from silk cocoons (TTSAM, China) following established methods.^13^^,^^15^ A 10% (w/v) silk fibroin electrospinning solution was prepared using a wet process to generate randomly structured matrices. To fabricate sponge-like silk fiber matrices (SFM), the silk constructs were immersed in 100% ethanol (Warner Graham Company, USA) for 45 min to induce full cross-linking, followed by thorough washing. The matrices were then frozen in deionized water in 6 cm culture dishes (Corning, New York, USA) and lyophilized for three days. Final SFM specimens were cut into uniform discs (4 mm diameter × 0.2 mm thickness) using biopsy dermal punches (Painful Pleasures, USA). Test compounds included three FDA-approved antiretroviral drugs—TFV (3 μM and 30 μM),^12^^,^^16^^,^^17^ emtricitabine (FTC, 7 μM and 70 μM) 18, 19, 20, and raltegravir (RAL, 2 μM and 20 μM)^21^^,^^22^—sourced from the NIH HIV Reagent Program. Rotenone (RTNN, 10 μM; Millipore Sigma)^23^ served as the positive control, while 0.1% dimethyl sulfoxide (DMSO; Millipore Sigma) was used as the vehicle and negative control. All compounds were dissolved in 0.1% DMSO at 1000 × stock concentrations and added to the culture medium at a final volume of 100 μL per 100 mL. Three biological replicates were tested per drug concentration, with DMSO-treated controls included in parallel. Each drug was tested at two concentrations in the culture media. MtT was evaluated in 3D-SFM cultures of oUSC and yUSC after 6 weeks, and in HKE293 and HepG2 monolayer cultures after 3 days.

The decision to utilize a 10-fold clinically relevant concentration (supraphysiological dose) of TFV in our *in vitro* 3D culture model is consistent with established methodologies in drug toxicology and safety studies. This approach is designed to deliberately stress the biological system and establish a comprehensive safe margin of exposure. By accelerating the detection of potential low-level, long-term toxic effects within a short *in vitro* timeframe, this higher concentration provides a robust assessment that would be challenging to achieve under purely physiological dosing conditions. The supraphysiological concentration serves as an *in vitro* proxy for worst-case clinical scenarios. These scenarios include, but are not limited to, high local tissue accumulation (a critical consideration given TFV's significant active intracellular uptake), impaired drug clearance resulting from renal dysfunction, or episodes of transiently elevated systemic levels due to unforeseen drug–drug interactions. Using this dose ensures that the model is sensitive enough to capture potential toxicity across the entire clinical safety spectrum. For all compounds tested, the clinically relevant concentrations were determined by referencing the reported maximum plasma concentration (*C*_max_) values derived from published pharmacokinetic/clinical studies or drug labels.^12^^,^^16^, ^17^, ^18^, ^19^, ^20^, ^21^, ^22^ When a supraphysiological dose was employed for other compounds, the fundamental rationale remained consistent: to effectively probe for off-target toxicity and precisely define the therapeutic safety window. This strategy explicitly accounts for potential pharmacokinetic/pharmacodynamic (PK/PD) discrepancies and limitations inherent to the simplified *in vitro* testing environment compared with the complex *in vivo* setting.

### Culture medium and supplements

Keratinocyte serum-free medium was supplemented with 5 ng/mL epidermal growth factor, 50 ng/mL bovine pituitary extract, 30 ng/mL cholera toxin, 100 U/mL penicillin, and 1 mg/mL streptomycin. Progenitor cell medium consisted of ¾ Dulbecco's modified Eagle's medium, ¼ Hamm's F12, 10% fetal bovine serum, 0.4 mg/mL hydrocortisone, 10^−10^ M cholera toxin, 5 ng/mL insulin, 1.8 × 10^−4^ M adenine, 5 mg/mL transferrin plus 2 × 10^−9^ M 3,39,5-triiodo-l-thyronine, 10 ng/mL epidermal growth factor, and 10% penicillin and streptomycin. All culture reagents were purchased from Gibco (Thermo Fisher Scientific, Waltham, Massachusetts, USA). Acetone, ethanol, methanol, isopropanol, phosphate-buffered saline (PBS), and other reagents were used as needed, with demineralized water utilized throughout.

### Collection, isolation, and culture of primary human urinary stem cells

Human urine samples (volume 100–300 mL) were collected from 6 healthy young male donors (aged 18–40 years) and 6 healthy elderly male donors (aged 65–80 years). Samples were centrifuged, and the resulting cell pellets were washed thoroughly with PBS. Cells were then plated onto culture dishes using USC medium. USCs at passage 3 were cultured at 37 °C in a humidified incubator containing 5% CO_2_ and utilized for all subsequent experiments. Human embryonic kidney cells (HEK293T, CRL-3216) and human hepatoblastoma cells (HepG2, HB-8065), obtained from the American Type Culture Collection, were used as cell line controls.

### Cell proliferation assays

Cell proliferation and viability were assessed at various time points after seeding oUSCs, yUSCs, HEK293 cells, and HepG2 cells on 3D-SFM in 96-well plates. Cell Counting Kit-8 (CCK-8 assay, Dojindo, Japan) was used according to the manufacturer's instructions, with absorbance measured at 450 nm using a microplate reader (MultiSkan FC, Thermo, USA).

### Live/dead assay

Cell viability of oUSCs, yUSCs, HEK293 cells, and HepG2 cells on 3D-SFM was assessed by a live/dead assay (Thermo Fisher) at day 3 and week 6. Calcein AM and EthD-1 were diluted in PBS to prepare 2 mM and 4 mM working solutions, respectively. Cell-seeded matrices were washed thoroughly with PBS and incubated with the staining solution at room temperature for 15 min. Following incubation, stained cell constructs were examined using confocal microscopy (Leica TCS-LSI, Leica Biosystems Inc., Buffalo Grove, Illinois, USA).

### Ultrastructure of 3D cultures

The surface morphology of the 3D-SFM was evaluated using a scanning electron microscope. The samples were first fixed in 2.5% glutaraldehyde and then dehydrated using a Leica EM CPD300 Critical Point Dryer (Leica Microsystems GmbH, Wetzlar, Germany). Subsequently, samples were mounted and sputter-coated with gold. Scanning electron microscopy imaging was performed using a FlexSEM 1000 microscope (Hitachi Medical Systems America Inc., Twinsburg, Ohio, USA) at accelerating voltages of 5–7 kV and working distances between 5.5 and 7 mm.

### Fluorescence staining for mitochondrial mass

Mitochondrial mass was assessed using MitoTracker Green FM (Thermo Fisher, Waltham, Massachusetts, USA), a green-fluorescent mitochondrial stain. Cells were washed with PBS, followed by incubation with 100 nM working solution at 37 °C for 15 min. After incubation, cells were washed again with PBS, and mitochondrial staining was visualized using confocal microscopy (Leica TCS-LSI, Leica Biosystems Inc., Buffalo Grove, Illinois, USA).

### Immunohistochemical assessment of mitochondrial superoxide dismutase and β-galactosidase activity staining

To evaluate mitochondrial superoxide dismutase 2 (SOD2) expression, cells from 3D-cultured matrices (oUSC-SFM, yUSC-SFM, HepG2-SFM, and HEK293-SFM) were detached using trypsin and seeded onto 8-well chamber slides. Cells were fixed in 4% paraformaldehyde for 30 min, permeabilized with 0.2% Triton X-100, and blocked with Dako protein block. Samples were then incubated at 4 °C overnight with a primary antibody against human SOD2 (Cell Signaling Technology, USA; diluted 1:50). Subsequently, cells were incubated at room temperature for 2 h with an Alexa Fluor 647-conjugated goat anti-mouse secondary antibody (Thermo Fisher Scientific, USA). Immunofluorescence was visualized using a Leica DM4000 B microscope (Leica Microsystems, Wetzlar, Germany). Staining for β-galactosidase activity was performed using a commercial kit (CS0030; MilliporeSigma, Burlington, Massachusetts, USA), following the manufacturer's instructions.

### Real-time PCR

To quantify the expression levels of cell senescence genes (p53, p21, and Rb) and mtDNA, mRNA expression in oUSC, yUSC, HEK293, and HepG2 on 3D-SFM was assessed using the Bio-Rad CFX connect Real-Time PCR Detection System. To determine mRNA expression in drug-treated USC within 3D cultures, mRNA was extracted using the RNeasy Mini Kit (Qiagen, Valencia) and reverse-transcribed to cDNA using the High-Capacity cDNA Reverse Transcription Kit (Thermo Fisher, USA). The remaining reagents and primers were identical to those used for real-time PCR for mtDNA. The PCR temperature cycling protocol included an initial denaturation at 50 °C for 2 min, followed by 95 °C for 10 min. Subsequent steps mirrored the real-time PCR for genes with normalization against GAPDH. The primer sequences are listed in Table S1.

### Western blotting analysis

Western blotting analysis was performed to evaluate the expression of mitochondrial complexes I–V, SOD2, KIM-1, CYP2E1, caspase-3, cleaved caspase-3, PGC1-α, TRF1, mtTFA, Nrf1, and Nrf2. Four types of cells, oUSC, yUSC, HepG2, and HEK293 cell lines in 3D SFM were loaded, separately, into 81-well molds provided by Micro-Tissues 3D Petri Dish (Sigma, USA). Cell samples were washed with PBS, harvested, and incubated for 30 min in the presence of 500 μL lysis buffer (Pierce, Rockford, Illinois, USA) supplemented with 1% protease/phosphatase inhibitor cocktail (Cell Signaling Technology, Danvers, Massachusetts, USA). Vertexing was performed every 5 min during incubation. After centrifugation, protein concentrations were determined using the Pierce™ BCA Protein Assay Kit. After protein separation on 12% SDS-PAGE gels, proteins were transferred to a PVDF membrane (Thermo Fisher) using Power Blotter (Thermo Fisher) for 7 min. The membrane was blocked for 1 h in PBS with 0.1% Tween 20 (PBST) containing 5% skim milk, washed with PBST, and incubated with primary antibodies (Table S2) at 4 °C overnight. Primary antibody dilutions were prepared in 5% bovine serum albumin. After extensive PBST washing, the membrane was exposed to secondary antibodies at room temperature for 1 h. The washed membrane was then treated with Immobilon ECL Ultra Western HRP Substrate (Millipore Sigma) and analyzed using an iBright™ CL1500 Imaging System.

### Telomerase activity assay

Telomerase activity levels were measured using the Telo TAGGG Telomerase PCR ELISA plus Kit (Roche Applied Science, Mannheim, Germany) according to the manufacturer's recommendations. HEK 293 cells were used as a positive control. Briefly, 4 × 10^4^ cells were collected at 6 weeks after trypsinization and washing with cold PBS. Telomerase added telomeric repeats (TTAGGG) in the kit to the 3′ end of the biotin-labeled synthetic P1-TS primer. The elongated products, together with the internal standard (IS) contained in the same reaction vessel, were amplified by PCR using the P1-TS primer and the anchor primer P2. The resulting products were divided into two aliquots, denatured, and hybridized separately with digoxigenin-labeled (DIG) detection probes specific for telomeric repeats and IS (P3-Std). The resulting products were immobilized on a streptavidin-coated microplate via the biotin label. Immobilized amplicons were detected with an antibody to digoxigenin conjugated to horseradish peroxidase (anti-DIG-HRP) and the sensitive peroxidase substrate TMB. Absorbance values were measured as the A450 nm reading against a blank (reference wavelength A690 nm) using a SpectraMax M5 Microplate Reader.

### Statistical analysis

Descriptive statistics were presented as mean ± standard deviation using OriginLab software version 2024. All data shown were derived from experiments that were independently repeated at least three times. For experiments with multiple treatments, such as different doses, one-way ANOVA with Dunnett's multiple comparisons to the control group (DMSO) or multiple unpaired *t*-tests were employed. For experiments with two groups, such as different time points, the Student's *t*-test was used for comparisons, with Bonferroni's multiple comparisons applied when appropriate. A *p*-value < 0.05 was considered statistically significant.

## Results

### Fabrication of 3D silk fiber matrix and drug toxicity assay

Scanning electron microscopy revealed distinct cell morphologies on the surface of the SFM. yUSCs and HEK293 cells exhibited a more rounded morphology, whereas oUSCs and HepG2 cells displayed a flattened appearance (Fig. S1A). Under high-power light microscopy, USCs were observed growing in grape-like clusters along the silk fibers at the edges of the SFM. After six weeks of culture, a greater number of yUSCs adhered to the silk fibers compared with oUSCs, which showed reduced attachment (Fig. S1B).

Live/dead staining over a 6-week culture period demonstrated that both oUSCs and yUSCs were evenly distributed and proliferated within the SFM, while HEK293 and HepG2 cells tended to aggregate, forming spherical or tissue-like structures (Fig. S1C). The numbers of yUSCs and oUSCs remained relatively stable from baseline to week 6 but declined significantly by week 8 compared with the initial seeding density. In contrast, HepG2 and HEK293 cell numbers increased approximately 10-fold within the first 2 weeks and then plateaued, as determined by the CCK-8 assay (Fig. S1D and S1E). These findings suggest that human primary USC in 3D culture more closely recapitulate *in vivo* conditions than established cell lines, characterized by the absence of both rapid proliferation and significant cell death over 6 weeks—features that make them particularly suitable for assessing mitochondrial function.

### Effects of antiretroviral drugs on mitochondrial function and viability of USCs in 3D cultures

**Figure 1:**
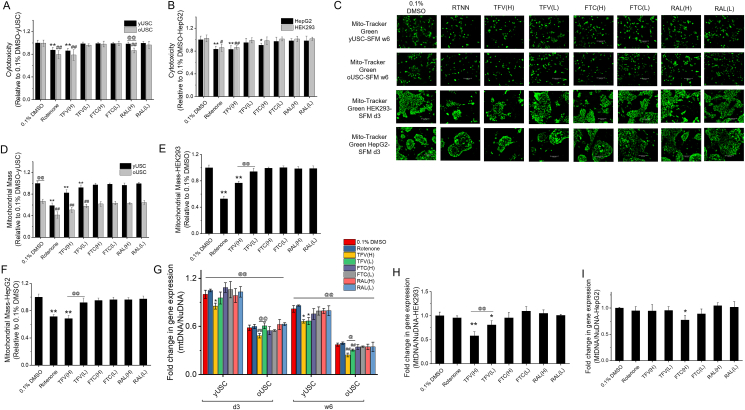
Cell viability, mitochondrial mass, and mitochondrial DNA copy number (mtCN) in oUSCs exposed to antiretroviral drugs. **(A, B)** Cell viability was assessed using the CCK-8 assay in USCs after 6 weeks of treatment and in HEK293 and HepG2 cells after 3 days. **(C)** MitoTracker Green staining of mitochondria in USCs at 6 weeks and in HEK293 and HepG2 cells at 3 days, visualized by confocal microscopy. **(D**–**F)** Quantitative analysis of mitochondrial mass based on MitoTracker Green fluorescence, expressed as an index relative to yUSCs treated with 0.1% DMSO. **(G)** mtDNA content in USCs was quantified by reverse transcription-PCR at 3 days and 6 weeks following drug exposure. Values were expressed as the ratio of mtDNA to nuclear DNA (NuDNA), normalized to yUSCs treated with 0.1% DMSO. **(H, I)** mtDNA content in HEK293 and HepG2 cells was assessed by reverse transcription-PCR at day 3, with results expressed as the mtDNA/NuDNA ratio. *n* = 6. Data were expressed as mean ± standard deviation. Student's *t*-test was used. ∗*p* < 0.05; ∗∗*p* < 0.01. ∗ versus 0.1% DMSO control in yUSCs; ^#^ versus 0.1% DMSO control in oUSCs.

Rotenone and high-dose TFV significantly reduced the viability of both oUSCs and yUSCs at 6 weeks, as well as HepG2 and HEK293 cells at day 3. Additionally, high-dose RAL markedly decreased oUSC viability at 6 weeks but had no significant effect on the other three cell types. High-dose FTC significantly reduced HepG2 viability at day 3 but did not affect the remaining cell groups (Fig. 1A and B). These findings suggest that rotenone and high-dose TFV exhibit cytotoxicity across all four cell types, whereas low-dose TFV, high-dose RAL, and FTC do not display cytotoxic effects under the tested conditions.

MitoTracker Green is a widely used method for assessing mitochondrial function by labeling and tracking mitochondria within stem cells.^24^^,^^25^ The mitochondrial mass index was significantly lower in oUSCs than in yUSCs under baseline conditions (0.1% DMSO) as well as across all treatment groups at 6 weeks, indicating that oUSCs intrinsically possess a lower mitochondrial mass than yUSCs (*p* < 0.01). Moreover, when normalized to baseline (yUSCs treated with 0.1% DMSO), the mitochondrial mass index of yUSCs was significantly reduced following treatment with both low- and high-dose TFV, as well as rotenone (Fig. 1C and D). In contrast, the mitochondrial mass index of HEK293 and HepG2 cells declined significantly only under high-dose TFV and rotenone treatment (Fig. 1C, E, F). These results suggest that both low- and high-dose TFV selectively reduce mitochondrial mass in primary human USCs, but not in established cell lines. Therefore, long-term 3D culture of USCs provides a more sensitive platform for detecting TFV-induced mitochondrial impairment at clinically relevant concentrations.

The genetic integrity of mtDNA is essential for maintaining normal cellular function.^26^^,^^27^ In oUSCs, mtCN was significantly lower than that in yUSCs under baseline conditions and across all treatment groups from day 3 to week 6. Additionally, mtCN levels in both oUSCs and yUSCs were significantly reduced at week 6 compared with their respective levels at day 3, indicating a time-dependent decline in mtCN associated with both antiretroviral drug exposure and donor age. Notably, high-dose TFV treatment led to a significant reduction in mtCN in both oUSCs and yUSCs at both 3 days and 6 weeks. A similar decline in mtCN was observed at 6 weeks in cells treated with clinically relevant doses of TFV (Fig. 1G). These findings suggest that long-term 3D culture over 6 weeks enhances the detection of TFV-induced mtCN depletion, particularly at therapeutic concentrations, compared with short-term culture. In HEK293 cells, TFV induced a dose-dependent reduction in mtCN at day 3, whereas in HepG2 cells, mtCN was decreased following high-dose FTC treatment for 3 days (Fig. 1H and I).

### SOD2 expression in 3D-SFM culture

**Figure 2:**
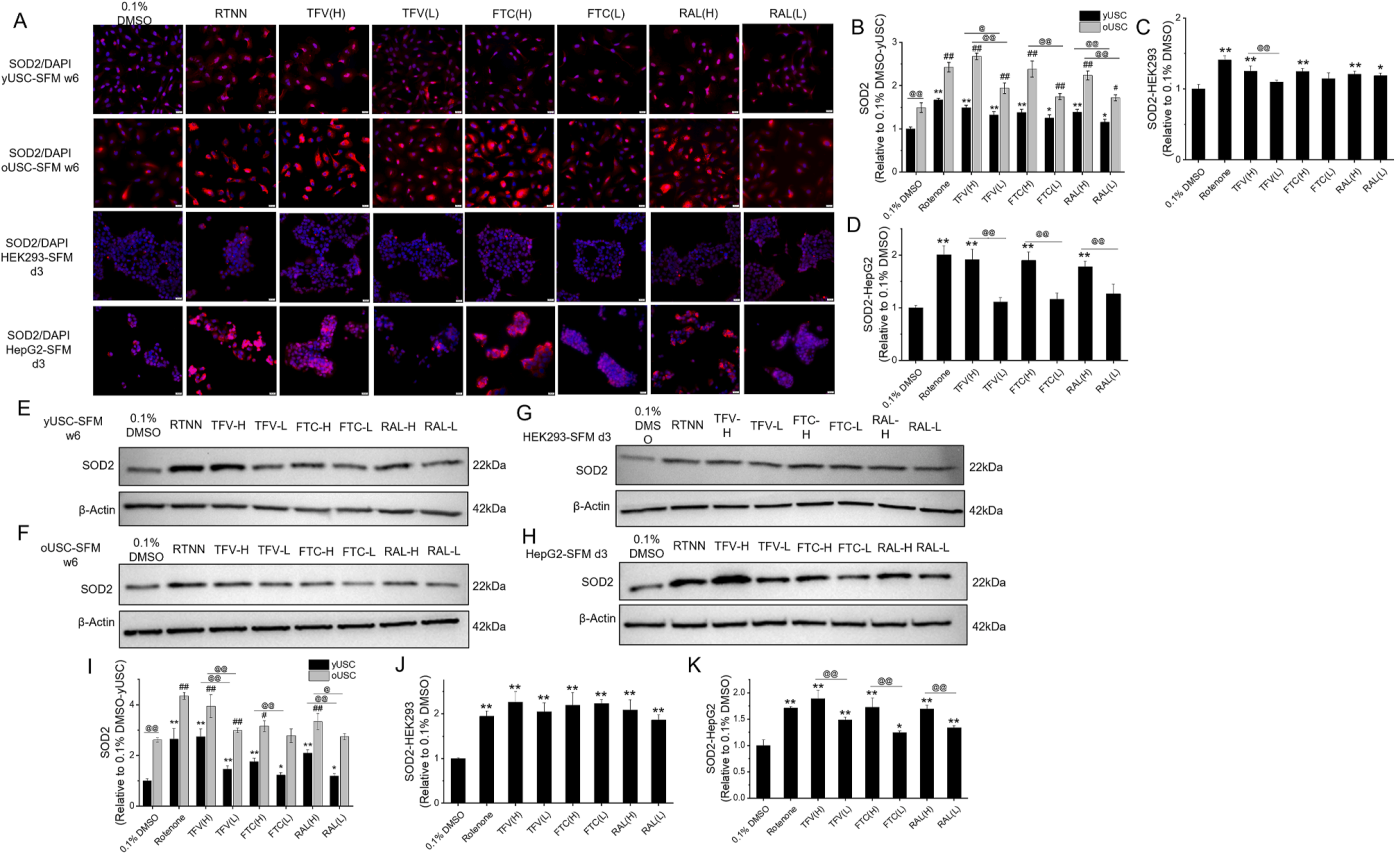
SOD2 expression in 3D-SFM cultures. **(A)** Double immunofluorescence staining for SOD2 (green) and DAPI (blue) was performed in yUSCs, oUSCs, HEK293 cells, and HepG2 cells. **(B**–**D)** Semi-quantitative analysis of immunofluorescence images shown in (A). SOD2 expression levels were normalized to the 0.1% DMSO control for yUSCs, HEK293 cells, and HepG2 cells. **(E**–**H)** Mitochondrial SOD2 protein levels were evaluated by Western blotting in USCs after 6 weeks of culture. **(I–K)** Densitometric analysis of Western blot bands shown in (E–H). SOD2 expression was quantified relative to the housekeeping protein β-actin for each cell type. *n* = 6. Data were expressed as mean ± standard error of the mean. Student's *t*-test was used. ∗*p* < 0.05; ∗∗*p* < 0.01. ∗ versus 0.1% DMSO control in yUSCs; ^#^ versus 0.1% DMSO control in oUSCs.

SOD2 plays a pivotal role in maintaining cellular redox homeostasis, particularly in mitigating mitochondrial oxidative stress.^28^ After 6 weeks of culture, SOD2 expression in USC was significantly increased under the following four conditions: i) in oUSCs compared with yUSCs at baseline, indicating an age-related up-regulation of SOD2 in oUSCs; ii) in oUSCs versus yUSCs across all drug treatment groups, suggesting drug-associated enhancement of SOD2 expression in oUSCs; iii) in both yUSCs and oUSCs following each drug treatment compared with their respective baselines, demonstrating that all tested drugs induced SOD2 expression in both cell types; and iv) in USCs treated with high-dose TFV and RAL compared with low-dose treatments, indicating a dose-dependent effect of these agents on SOD2 up-regulation (Fig. 2A, B, E, F, I). Comparable trends were observed in HEK293 and HepG2 cells after 3 days of treatment. Specifically, SOD2 expression was significantly increased: i) in each drug-treated group relative to untreated controls; and ii) in HepG2 cells treated with high versus low doses of all three drugs, indicating a dose-dependent effect in HepG2 but not in HEK293 cells (Fig. 2A, C, D, G, H, J, K). Collectively, these results indicate that mitochondrial oxidative stress is more pronounced in oUSCs than in yUSCs under long-term 3D culture. Moreover, all tested antiretroviral agents can induce mitochondrial oxidative stress in all four cell types, with clear dose-dependent effects.

### Dysfunctional electron transport chain (ETC) complexes I–V in 3D-SFM cultures treated with antiretroviral drugs over time

**Figure 3:**
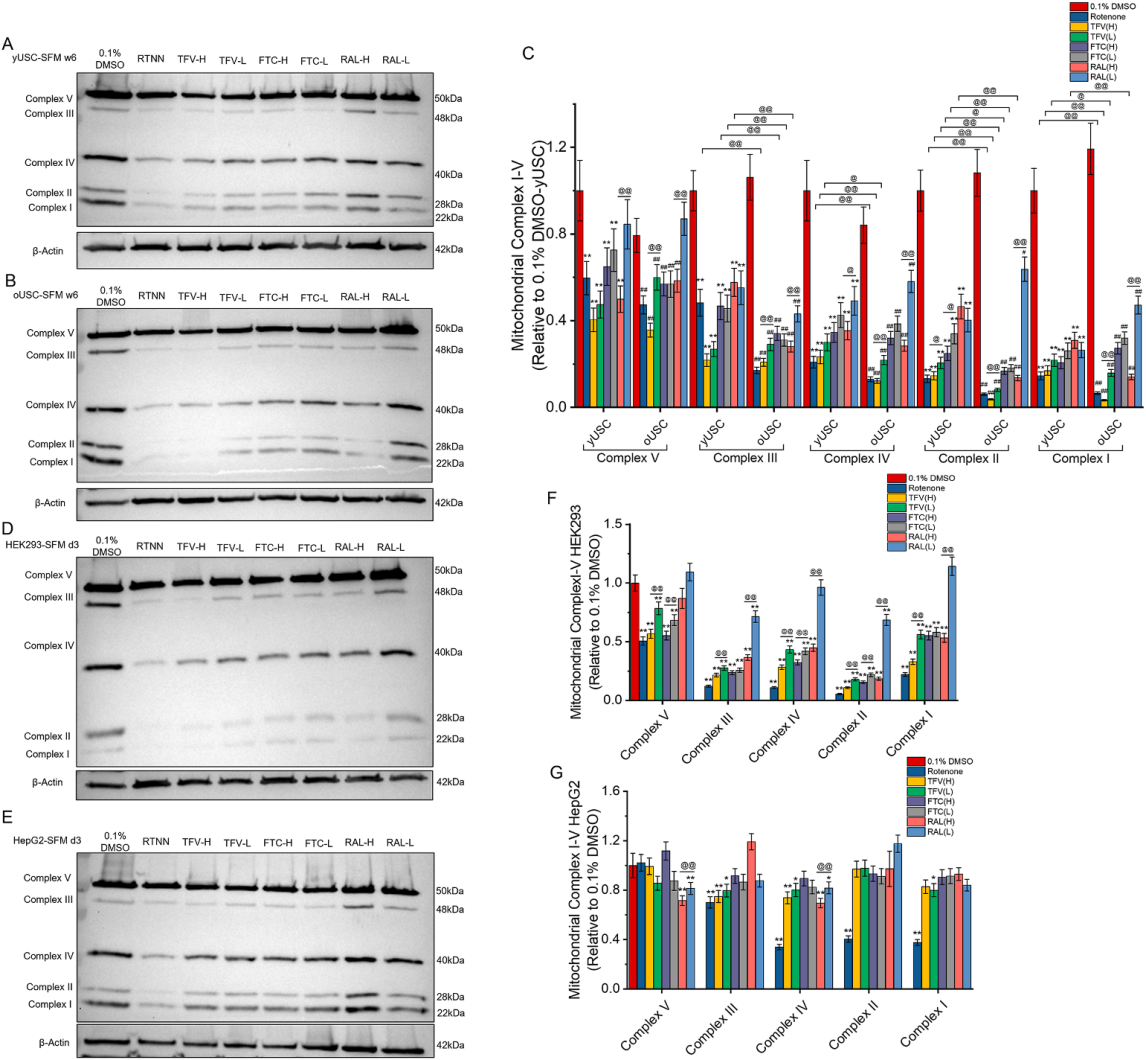
Dysfunctional ETC complexes I–V in 3D SFM cultures treated with antiretroviral drugs over time. **(A, B)** Western blotting analysis of ETC complex I (NDUFB8), complex II (SDHB), complex III (UQCRC2), complex IV (MTCOXI), and complex V (V-ATP5A) in yUSCs and oUSCs after 6 weeks of treatment with antiretroviral drugs at low and high doses. **(C)** Semi-quantitative analysis of the Western blot bands shown in (A, B). Band intensities were normalized to the housekeeping protein β-actin. Protein expression levels of ETC complexes I–V were expressed relative to yUSCs treated with 0.1% DMSO. Sensitivity to drug-induced changes in oUSCs was compared to that in yUSCs. **(D, E)** Western blotting analysis of ETC complexes I–V in HEK293 and HepG2 cells after 3 days of treatment with high and low doses of the indicated drugs. **(F, G)** Semi-quantitative analysis of the Western blot bands in (D, E), with protein levels normalized to β-actin. *n* = 6. Data were expressed as means ± standard deviation. Student's *t*-test was used. ∗*p* < 0.05; ∗∗*p* < 0.01. ∗ versus 0.1% DMSO control in yUSCs; ^#^ versus 0.1% DMSO control in oUSCs.

Mitochondria act as key signaling organelles that govern stem cell fate and function.^29^ In this study, we observed that all tested drugs significantly reduced the protein levels of ETC complexes I–V in both yUSCs and oUSCs, with the exception of low-dose RAL, which selectively affected complex V and complex III in both cell types. Notably, TFV induced a dose-dependent reduction in the levels of complexes I–V in oUSCs, but not in yUSCs, after 6 weeks of treatment. At baseline, there were no significant differences in ETC complex protein levels between yUSCs and oUSCs. However, following drug exposure, oUSCs exhibited significantly lower levels of complexes I–IV compared with yUSCs, indicating that oUSCs are more susceptible to drug-induced mitochondrial damage—particularly from TFV (Fig. 3A–C). In HEK293 cells treated for 3 days, TFV caused a dose-dependent decrease in the protein levels of complexes I–V, whereas high-dose RAL led to a reduction in complexes I–IV. In HepG2 cells, TFV treatment reduced the protein levels of complexes III and IV, while RAL caused a dose-dependent decrease in complexes IV and V (Fig. 3D–G). Collectively, these findings indicate that TFV induces a dose-dependent reduction in the protein levels of ETC complexes I–V in oUSCs, with a more pronounced impact on oUSCs compared with yUSCs, particularly affecting complexes I–IV.

### Expression ratio of cleaved caspase-3/caspase-3 to apoptosis protein increased in 3D-SFM cultures

**Figure 4:**
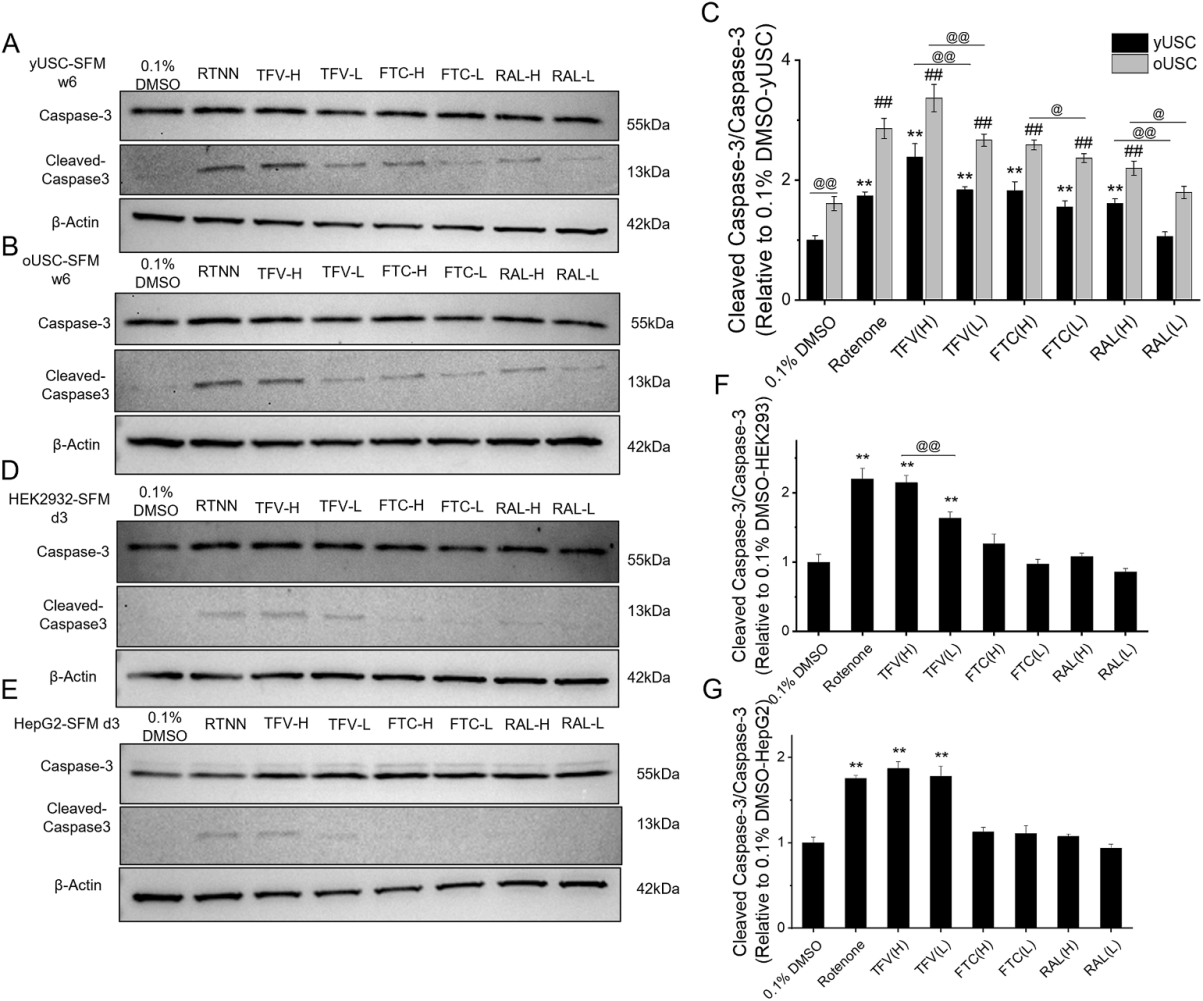
Increased expression ratio of cleaved caspase-3 to caspase-3 in 3D-SFM cultures. **(A, B)** Protein levels of cleaved caspase-3 and total caspase-3 were evaluated by Western blotting analysis in yUSCs and oUSCs after 6 weeks of drug treatment. **(C)** Semi-quantitative analysis of the cleaved caspase-3/caspase-3 ratio from (A, B), expressed relative to yUSCs treated with 0.1% DMSO as baseline. **(D, E)** Western blotting analysis of cleaved caspase-3 and caspase-3 in HEK293 and HepG2 cells after 3 days of drug exposure. **(F, G)** Semi-quantitative analysis of band intensities from (D, E), normalized to the housekeeping protein β-actin for each cell type. *n* = 6. Data were expressed as mean ± standard deviation. Student's *t*-test was used. ∗*p* < 0.05; ∗∗*p* < 0.01. ∗ versus 0.1% DMSO control in yUSCs; ^#^ versus 0.1% DMSO control in oUSCs.

Mitochondria are central players in the regulation of apoptosis.^30^ Activation of caspase-3 and cleavage of caspase 3 are critical steps in the apoptotic process, ensuring that cell death occurs in a controlled and regulated manner.^31^ At baseline, the ratio of cleaved caspase-3 to total caspase-3 protein levels was significantly higher in oUSCs compared with yUSCs, indicating an age-associated increase in apoptotic priming. Upon treatment, this ratio increased significantly in both yUSCs and oUSCs for all drugs except low-dose RAL. Moreover, both TFV and RAL induced a dose-dependent elevation in the cleaved caspase-3/caspase-3 ratio (Fig. 4A–C). In HEK293 and HepG2 cells, both rotenone and TFV elevated the cleaved caspase-3/caspase-3 ratio after 3 days of treatment, with TFV exhibiting a dose-dependent effect in HEK293 cells (Fig. 4D–G). These findings indicate that oUSCs undergo greater levels of apoptosis than yUSCs under baseline and drug-treated conditions. Furthermore, TFV exacerbated apoptosis in a dose-dependent manner in USCs during long-term 3D culture. Compared with HEK293 and HepG2 cells at 3 days, USCs cultured in 3D-SFM for 6 weeks demonstrated enhanced susceptibility to chronic, antiretroviral agent-induced apoptotic stress.

### Expression of senescence and stemness in 3D-SFM cultures

**Figure 5:**
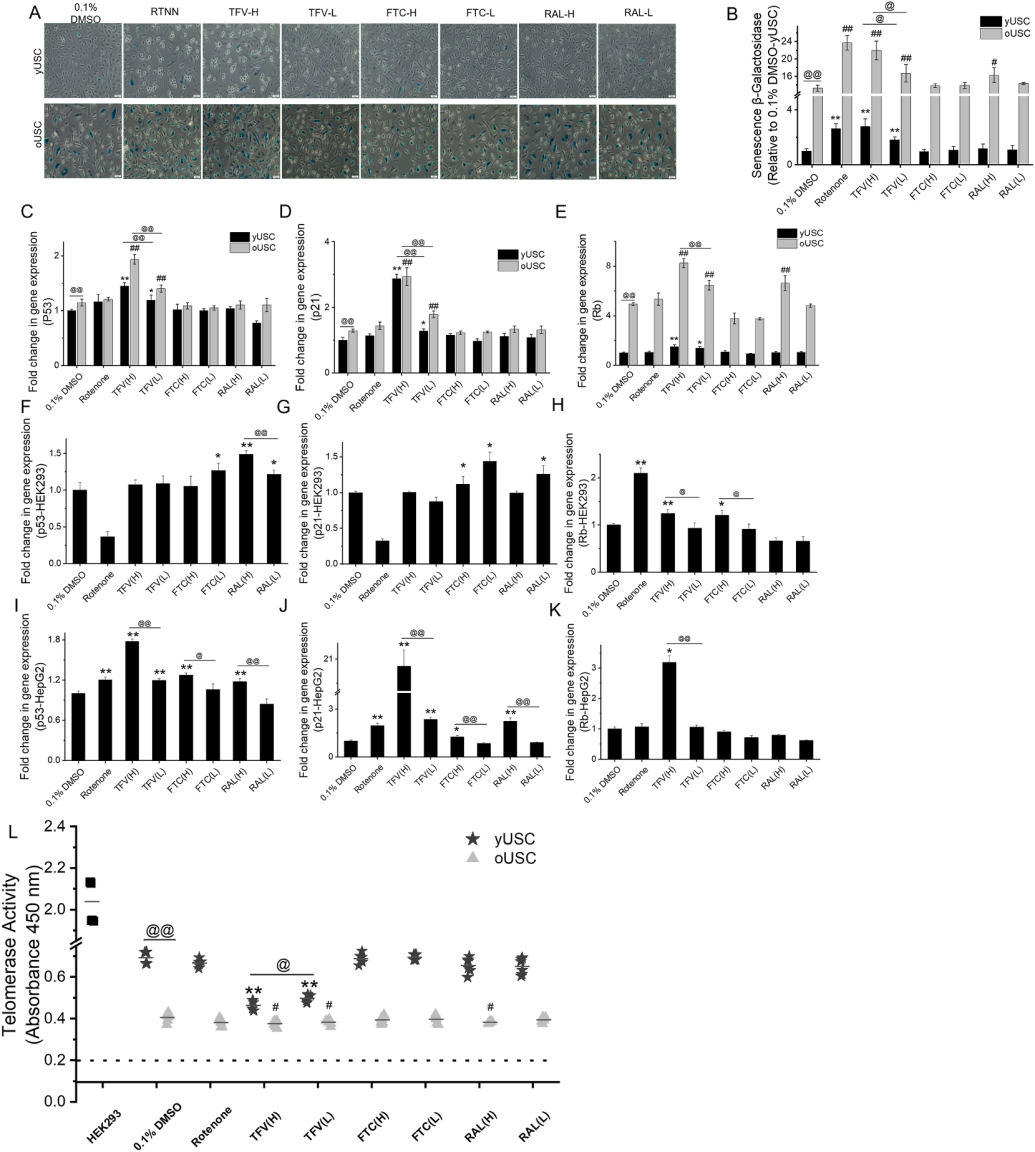
Expression of senescence and stemness in 3D-SFM cultures. **(A)** Senescence-associated β-galactosidase (β-gal) staining was performed in yUSCs and oUSCs after 6 weeks of culture. **(B)** Semi-quantitative analysis of β-gal staining intensity in (A), with yUSCs set as the baseline. **(C**–**E)** Expression of senescence-related genes (p53, p21, and Rb) in yUSCs and oUSCs at 6 weeks, assessed by quantitative PCR. **(F–K)** Expression of p53, p21, and Rb in HEK293 and HepG2 cells at 3 days, evaluated by quantitative PCR. **(L)** Telomerase activity in yUSCs and oUSCs following drug treatment for 6 weeks. HEK293 lysate was used as a representative positive control for telomerase activity. *n* = 6. Data were expressed as mean ± standard deviation. Student's *t*-test was used. ∗*p* < 0.05; ∗∗*p* < 0.01. ∗ versus 0.1% DMSO control in yUSCs; ^#^ versus 0.1% DMSO control in oUSCs.

Mitochondria play an important role in the aging process regarding cell senescence and regeneration potential, and mitochondrial dysfunction is a well-established hallmark of aging.^32^ At baseline, β-galactosidase (β-gal) activity, a classic marker of cellular senescence, was significantly higher in oUSCs compared with yUSCs. TFV treatment led to a dose-dependent increase in β-gal activity in both cell types, whereas high-dose RAL increased β-gal activity in oUSCs but not in yUSCs (Fig. 5A and B). The p53, p21, and retinoblastoma (Rb) genes are key players in the regulation of cell cycle progression and are closely associated with aging.^33^ Quantitative PCR analysis revealed that baseline mRNA levels of p53, p21, and Rb were elevated in oUSCs compared with yUSCs after 6 weeks in culture. Both low- and high-dose TFV treatment further increased the expression of all three genes in both cell types. Moreover, TFV induced a dose-dependent up-regulation of p53 and p21 in both yUSCs and oUSCs, and of Rb specifically in oUSCs (Fig. 5C–E). In HEK293 cells, rotenone and high-dose TFV increased Rb mRNA levels after 3 days, but did not significantly alter p53 or p21 expression. In contrast, in HepG2 cells, rotenone, TFV, high-dose FTC, and high-dose RAL up-regulated p53 and p21, whereas Rb expression was significantly increased only by high-dose TFV (Fig. 5F–K).

At baseline, telomerase activity in oUSCs was significantly lower than that in yUSCs (*p* < 0.01). TFV treatment significantly reduced telomerase activity in both oUSCs and yUSCs (*p* < 0.05), with a more pronounced decrease observed in oUSCs (*p* < 0.01). Moreover, TFV exhibited a dose-dependent inhibitory effect on telomerase activity in oUSCs (Fig. 5L). These findings suggest that USCs offer distinct advantages over immortalized cell lines (HEK293 and HepG2) for investigating cellular senescence and telomerase dynamics. Specifically, TFV promotes cellular senescence and reduces telomerase activity in a dose-dependent manner. Additionally, high-dose RAL was found to exacerbate senescence in oUSCs during long-term (6-week) 3D culture.

### KIM-1 and CYP2E1 protein expression in 3D-SFM cultures

**Figure 6:**
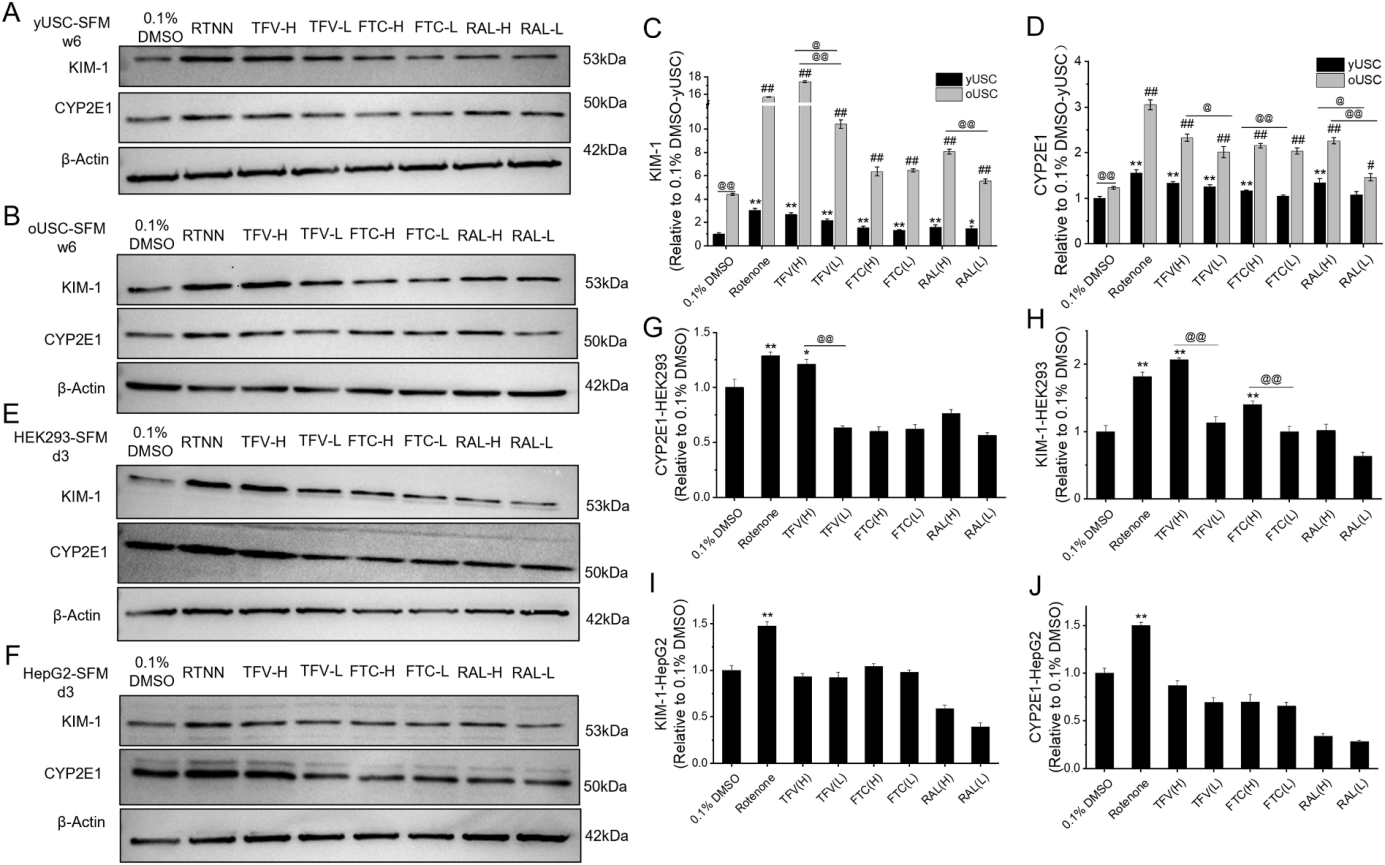
KIM-1 and CYP2E1 protein expression in 3D-SFM cultures. **(A, B)** Western blotting analysis of KIM-1 and CYP2E1 protein levels in yUSCs and oUSCs after 6 weeks of culture. **(C, D)** Semi-quantitative analysis of KIM-1 and CYP2E1 expression corresponding to (A, B). Protein expression levels are shown as indices relative to yUSCs treated with 0.1% DMSO. **(E, F)** Western blotting analysis of KIM-1 and CYP2E1 protein levels in HEK293 and HepG2 cells after 3 days of drug exposure. **(G**–**J)** Semi-quantitative analysis of KIM-1 and CYP2E1 expression corresponding to (E, F). Band intensities were normalized to the housekeeping protein β-actin. *n* = 6. Data were expressed as mean ± standard deviation. Student's *t*-test was used. ∗*p* < 0.05; ∗∗*p* < 0.01. ∗ versus 0.1% DMSO control in yUSCs; ^#^ versus 0.1% DMSO control in oUSCs.

KIM-1 serves as a predictive marker for the rate of kidney disease progression.^34^ CYP2E1 is an essential enzyme involved in drug metabolism, particularly in the oxidation of drugs and xenobiotics.^35^ After 6 weeks of 3D-SFM culture, baseline protein levels of KIM-1 and CYP2E1 were significantly higher in oUSCs compared with yUSCs (*p* < 0.01). Treatment with all tested drugs increased the expression of both KIM-1 and CYP2E1 in yUSCs and oUSCs, with the exception of low-dose FTC. TFV induced a dose-dependent increase in KIM-1 expression in both cell types and significantly elevated CYP2E1 levels in oUSCs (Fig. 6A–D). At the 3-day time point, TFV also caused a dose-dependent increase in both KIM-1 and CYP2E1 protein levels in HEK293 cells. In contrast, only rotenone elevated the expression of both proteins in HepG2 cells (Fig. 6E–J).

### TFV-mediated telomere-mitochondrial injury in oUSCs

**Figure 7:**
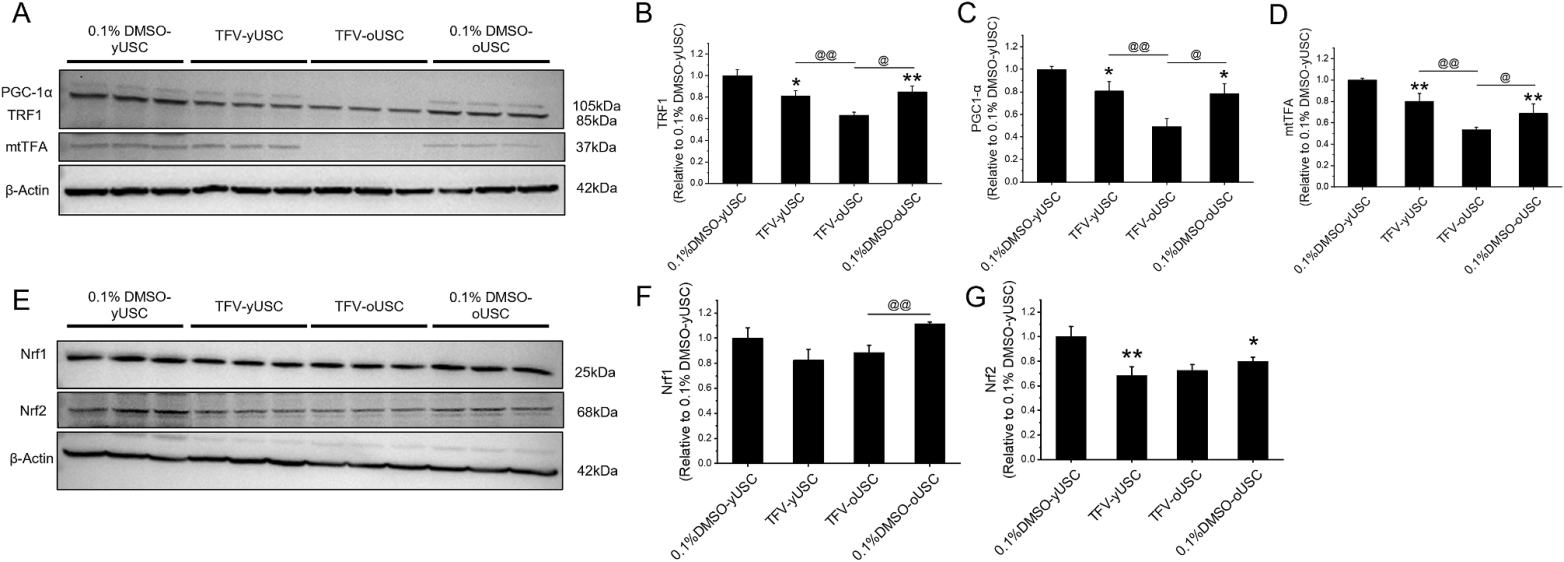
Low-dose TFV inhibits PGC-1α expression and induces mitochondrial toxicity in oUSCs in 3D culture. **(A)** Western blotting analysis of TRF1, PGC-1α, and mtTFA protein levels in oUSCs and yUSCs after 6 weeks of culture. **(B**–**D)** Semi-quantitative analysis of (A) shows a significant reduction in TRF1, PGC-1α, and mtTFA expression in oUSCs compared with yUSCs at 6 weeks. **(E)** Western blotting analysis of Nrf1 and Nrf2 protein levels in oUSCs and yUSCs after 6 weeks. **(F, G)** Semi-quantitative analysis of (E) reveals significant reductions in Nrf2 expression in oUSCs compared with yUSCs, and in Nrf1 expression in TFV-treated oUSCs versus baseline. *n* = 6. Data were expressed as mean ± standard deviation. Student's *t*-test was used. ∗*p* < 0.05; ∗∗*p* < 0.01. Control is yUSCs in 0.1% DMSO. ∗ versus 0.1% DMSO control in yUSCs; ^#^ versus 0.1% DMSO control in oUSCs.

Protein levels of TRF1, PGC-1α, and mtTFA were significantly reduced under three comparative conditions: i) in oUSCs versus yUSCs at baseline after 6 weeks of culture; ii) in both yUSCs and oUSCs treated with therapeutic (low) doses of TFV compared with their respective baseline controls; and iii) in oUSCs versus yUSCs following TFV treatment (Fig. 7A–D). In addition, Nrf1 protein expression was significantly reduced in TFV-treated oUSCs compared with untreated oUSCs at baseline. Nrf2 expression was also significantly decreased in two contexts: i) in oUSCs compared with yUSCs at baseline, and ii) in yUSCs treated with TFV compared with untreated yUSCs (Fig. 7E–G). Notably, there was no significant difference in Nrf1 expression between untreated oUSCs and yUSCs, whereas Nrf2 expression was consistently lower in oUSCs at baseline. Collectively, these findings suggest that Nrf2 may play a central regulatory role in mediating the increased susceptibility of oUSCs to TFV-induced mitochondrial dysfunction, as compared with yUSCs.

## Discussion

Despite improved healthcare leading to increased longevity, renal function declines with age, elevating the risk of drug-induced renal injury, including renal failure. Renal MtT is a common type of renal injury that can potentially progress to chronic and acute renal diseases,^36^ even end-stage renal disease if not promptly addressed.^37^ However, detecting drug-induced renal toxicity remains a major challenge due to the lack of physiologically relevant and predictive models. In this study, we demonstrated that age-associated, drug-induced renal MtT can be accurately detected using USCs from aged donors cultured within a well-characterized silk fibroin matrix. Using TFV as a representative nephrotoxic agent, we validated this 3D model for its sensitivity and specificity. This platform holds promise for broader application in assessing MtT induced by other drug classes, including anti-cancer agents, anti-hypertensive medications, and beyond.

Silk matrix is a promising material for creating 3D models to test renal toxicity due to several key properties.^11^^,^^12^ Its close resemblance to the extracellular matrix of human tissues provides a biomimetic environment for renal cells to thrive, while its biocompatibility minimizes the risk of adverse reactions, making it suitable for long-term implantation. Furthermore, the tunable mechanical properties of silk allow for accurate representation of different renal tissue stiffnesses, and its controllable pore sizes facilitate nutrient and waste exchange, as well as cell infiltration and growth. Silk's versatility extends to its processability into various 3D structures, enabling the creation of complex renal models with specific anatomical features. In addition, its surface chemistry can be modified to promote cell adhesion and proliferation, leading to the formation of functional tissue-like constructs. Importantly, silk can serve as a carrier for drug delivery, enabling controlled release of therapeutic agents to target specific renal cell populations. Given its biocompatibility, mechanical adaptability, structural versatility, and potential for drug delivery, coupled with its relatively low cost and abundance as previously reported 11, 12, 13, 14, this study further demonstrated that the silk matrix provides a robust and versatile platform for developing accurate and predictive models of age-related renal toxicity, ultimately accelerating the development of new therapeutic strategies.

**Figure 8:**
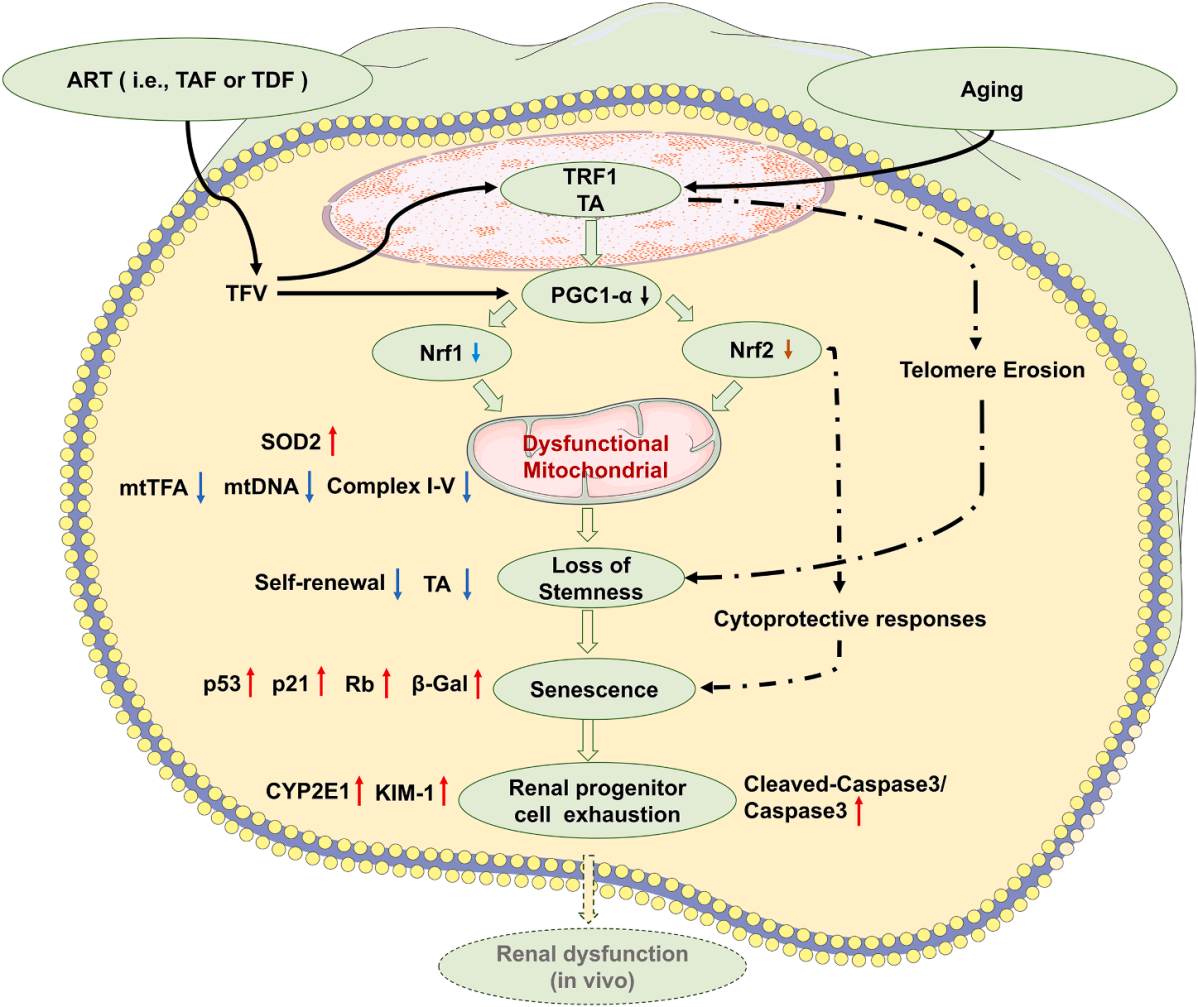
The functionality of oUSCs in the context of antiretroviral agents such as TFV. Upon entering USC, TFV induces a significant reduction in telomere parameters (TRF1 and telomerase activity proteins), which subsequently leads to a reduction in PGC1-α expression. This reduction in PGC1-α initiates mitochondrial dysfunction characterized by decreased mtTFA protein expression, increased SOD2 expression, decreased mtDNA content, and disruption of ETC complexes I–V. These changes result in a decrease in USC telomerase activity, indicating a loss of stem cell properties, and an increase in aging-related genes and markers, including β-Gal. In addition, increased levels of KIM-1 protein, CYP2E1, and cleaved-caspase3/caspase3 indicate cellular injury, reduced drug metabolism capacity, and the initiation of apoptosis in renal progenitor cells. Notably, the effect of TFV on oUSCs is exacerbated by their telomere-mitochondrial dysfunction compared with yUSCs.

Long-term or lifelong medications, such as antiretroviral agents, can induce MtT in multiple organs, including the kidney. These effects may be more pronounced in older individuals, leading to kidney damage. Drug-induced renal MtT can occur in somatic renal tubule epithelial cells. In this study, we found that telomere damage and MtT occurred in oUSCs compared with yUSCs, and were exacerbated by exposure to antiretroviral agents, particularly TFV, indicating that aging plays a critical role in the telomere–mitochondrial axis of renal stem cells. oUSCs had more serious drug-induced MtT injury than yUSCs, as evidenced by decreased telomerase activity, TRF, and mitochondrial mass, increased reactive oxygen species production, reduced complex I–V levels, and perturbations in mtDNA content and cell dysfunction. These data indicate that TFV-induced renal telomere–mitochondrial dysfunction may be more significant in older individuals due to the increased susceptibility of renal stem cells to MtT with aging. Furthermore, our study demonstrated that TFV treatment down-regulated the expression of TRF1, PGC-1α, mtTFA, Nrf2, and Nrf2, suggesting that the dysfunction of the telomere–mitochondrial axis plays a critical role in the development of TFV-induced MtT in oUSCs. The present study indicates that the PGC-1α pathway may be a potential therapeutic strategy for preventing or mitigating TFV-induced kidney damage (Fig. 8).

Drug-induced MtT and drug-induced cytotoxicity are two different types of cell injury, although they can overlap. Drug-induced MtT is a condition in which the mitochondria, the power plants of the cell, are damaged. This can lead to cell dysfunction, senescence, and death. Drug-induced cytotoxicity is a condition in which cells are damaged or killed by drugs through a variety of mechanisms, including damage to the cell membrane, DNA, or proteins. Drug-induced MtT is sometimes reversible, while drug-induced cytotoxicity is usually not. It is important to study drug-induced MtT early to prevent serious consequences. In this study, we built on our previous research and leveraged the advantages of the 3D USC-SFM culture system. We combined it with the use of three different antiretroviral drugs to simulate the scenario of combination antiretroviral therapy, which is common in HIV treatment. When USCs were treated with these agents, 95% of cells did not die, while MtT increased in time, particularly with low-dose TFV. We compared this approach with two cell lines commonly used in toxicity testing, HEK293 cells and HepG2 cells. Our goal was to delve deep into the chronic MtT of antiretroviral agents in renal stem cells and further investigate the mechanisms that might be involved. MtT is often overlooked in clinical settings because it lacks specific symptoms. Studying drug-induced MtT is important because it can help us to better understand, prevent, and treat its downstream effects.

Current drug toxicity models are mainly designed to test toxicities in different somatic cells. It is a novel invention to assess stem cell MtT using a 3D culture system for toxicological hazard^38^, ^39^, ^40^ and drug assessment.^41^, ^42^, ^43^, ^44^ Renal stem cells possess the unique ability to develop into specialized cell types to replace cells and renal tissues that have been damaged or lost. When renal stem cells are injured by toxicants, the kidney might face serious problems with the loss of tissue repair capacity, leading to kidney failure. Stem cells represent the most sensitive screening model, which can detect toxicity that may otherwise remain unnoticed.^45^ Currently, there are no *in vitro* models available to test tissue-specific stem cell toxicity because invasive approaches are required to obtain these stem cells. Human primary USCs 46, 47, 48, as renal progenitors first described by our team, are easily accessible and possess robust renewal capacity for tissue regeneration.^46^, ^47^, ^48^, ^49^, ^50^, ^51^, ^52^, ^53^ We also develop USC 3D models for testing drug-induced, delayed MtT^11^^,^^12^^,^^54^ and NT.^55^ Specifically, older antiretroviral drugs such as 3′-dideoxycytidine (ddC) and TDF significantly inhibited cell growth and resulted in MtT in USCs in 3D models.^11^^,^^12^ The present study extends the previous studies and moves towards addressing the validation of this method as a novel and useful tool to detect MtT of USCs. HEK293 is a commonly used cell type for renal toxicity^56^ or MtT.^57^ Despite being commonly used for drug testing, human cell lines (*i.e.*, HEK293 and HepG2) are not primary stem cells. Neither is suitable for a 3D culture system, as they grow too fast to accurately measure MtT. Our data revealed that USCs were superior to the HEK293 cell line: i) USCs in 3D culture could maintain stable cell survival and mitochondrial replication, compared with HEK293 cells, which is good for MtT assessment; ii) USCs were more sensitive compared with HEK293 cells and HepG2 cells exhibiting a reduction in mitochondrial mass, disrupted mitochondrial electron respiratory chain Complex I–V functionality, changes in oxidative stress, and alterations in cytochrome CYP2E1 enzyme expression under the influence of TFV. These cell lines do not accurately reflect the MtT of the drug in terms of mtDNA, apoptosis, and aging-related genes, due to their immortalized nature and continuous proliferation. This data suggests that USCs are an optimal source to test MtT for drug screening.

As individuals age, the mitochondria naturally become less efficient and produce more reactive oxygen species. This mitochondrial dysfunction is a hallmark of aging in many cell types, including renal stem cells. However, the mechanisms of drug-induced MtT, specifically TFV-induced MtT in oUSC, are more complex and need to be further studied. Our previous studies have shown that USCs exhibit weakened stemness and senescence with aging,^58^ and inhibited cell growth and mitochondrial dysfunction when exposed to antiretroviral agents.^11^^,^^12^ In this study, we found that: i) Mitochondrial function was declined in oUSCs compared with yUSCs; ii) Antiretrovirals, and particularly TFV, induced MtT in yUSCs; iii) Mitochondrial function was more significantly reduced in oUSCs than yUSCs when treated with TFV cultured in 3D suspension culture medium for an extended period of time. These findings suggest that mitochondrial dysfunction is a hallmark of aging in renal stem cells and that TFV exposure can exacerbate this dysfunction. This is particularly concerning for oUSCs, which already have reduced mitochondrial function compared with yUSCs. Our findings suggest that renal stem cells from older adults may be more susceptible to drug-induced MtT than those from younger adults. This could have implications for the design of antiretroviral agent regimens for older adults.

To evaluate the mitochondrial activities in oUSCs in 3D-SFM culture, we compared oUSCs to yUSCs six weeks after exposure to individual antiretroviral agents. We found that oUSCs had a reduced mitochondrial mass and mtDNA compared with yUSCs. This suggests that with increasing age, the number of mitochondria and mtDNA in renal stem cells progressively decreases, consistent with prior research.^59^ In addition, at six weeks, oUSCs showed higher mitochondrial SOD2 levels compared with yUSCs, indicating increased levels of mitochondrial oxidative stress in oUSCs relative to yUSCs. However, interestingly, when it came to the functionality of the mitochondrial ETC Complex I–V, there were no significant differences between oUSCs and yUSCs. We observed that oUSCs had higher levels of cleaved caspase-3/caspase-3 compared with yUSCs at six weeks, signifying more severe cell apoptosis in oUSCs. It is worth noting that reports in the literature have indicated a close association between mitochondrial function and the level of cellular apoptosis,^30^ which aligns with our experimental results. Some research has suggested that mitochondrial dysfunction and cell senescence are interconnected hallmarks of aging.^60^ In our study, we discovered that oUSCs exhibited higher levels of β-galactosidase expression compared with yUSCs. Furthermore, at six weeks, mRNA expression of p53-p21-Rb in oUSCs was higher than in yUSCs, indicating that aging-related increased senescence in oUSCs relative to yUSCs, consistent with most aging-related research.^61^^,^^62^ Nevertheless, the causal relationship between mitochondrial dysfunction and aging in this context still requires further investigation. The inability of aged stem cells to maintain quiescence during aging directly contributes to the loss of stemness.^63^ In our study, we found that the telomerase activity in oUSCs was significantly reduced compared with yUSCs, suggesting a decrease in stemness in oUSCs, in line with previous research. Decreased telomerase may also contribute to the increased senescence. We also observed increased levels of KIM-1 and decreased levels of cytochrome CYP2E1 in oUSCs, indicating a decline in drug metabolism capacity and increased vulnerability of cells.

To elucidate TFV-induced telomere-mitochondrial dysfunction in renal progenitor cells, we examined the role of PGC-1α in these cells maintained as a 3D culture for six weeks. TRF1 is an essential component of the telomeric protective complex or shelterin.^64^^,^^65^ In this study, we observed that the protein levels of TRF1 and PGC-1α in oUSCs significantly decreased compared with yUSCs, coupled with the reduced telomerase activity in oUSCs and the increased expression of the p53 gene as well. This suggests an association of PGC-1α down-regulation with TFV-induced telomere dysfunction and senescence in oUSCs. In addition, the previous studies showed that PGC-1α induced and co-activated Nrf-1/2 transcription, which initiated the transcription of mtTFA and genes related to the electron transport chain, oxidative phosphorylation (OXPHOS), and mitochondrial genome replication and transcription, promoting mitochondrial homeostasis and oxidative phosphorylation.^66^ In the present study, we observed a decrease in Nrf2 and mtTFA expression in oUSCs compared with yUSCs after treatment with TFV, while Nrf1 expression significantly decreased in TFV-treated oUSCs compared with oUSC DMSO-treated baseline, indicating that oUSCs may induce dysfunction of the telomere–mitochondrial axis via the PGC-1α pathway. This study suggests that telomere dysfunction activates p53-mediated cellular growth arrest, mitochondrial dysfunction, senescence, and apoptosis, with p53 binding to and repressing PGC-1α in renal progenitor cells in old adult after TFV treatment.

While our study demonstrated drug-induced perturbations in overall mitochondrial function and key markers of oxidative stress and apoptosis, a limitation is the absence of direct assessment of mitophagy—the selective degradation of damaged mitochondria. Specifically, the inclusion of upstream regulatory markers like PINK1 and Parkin would have provided a more detailed understanding of the mechanism by which the USCs attempt to clear the damaged mitochondria.^67^^,^^68^ Future work will incorporate these specific mitophagy markers to fully delineate the role of mitochondrial quality control in response to chronic drug exposure.

Either TDF or TAF can cause renal impairment, although TAF has been reported to do so less often than TDF.^69^ Whether or not TAF accumulates MtT in old adults over time is still unclear. However, our *in vitro* study demonstrated that TFV, the active form of TDF and TAF, can lead to mitochondrial dysfunction, cellular senescence, apoptosis, and cellular damage in oUSCs. Furthermore, we found that therapeutic-dose TFV decreased Nrf1 expression in oUSCs, which may contribute to the increased susceptibility of oUSCs to TFV-induced damage. This suggests that older adults may be at increased risk of developing MtT and other adverse effects from TDF and TAF, and that Nrf1 may play a role in this increased vulnerability.

Since current antiviral therapy involves the combination use of several (typically two or three) antiretroviral drugs for efficient antiretroviral treatment,^70^ we included two common antiretroviral drugs, FTC and RAL, in our experiments to determine whether other drugs also exhibit MtT. We found that both RAL and FTC could induce mitochondrial oxidative stress, disrupt the functionality of the mitochondrial electron respiratory chain complex I–V, induce apoptosis, increase the expression of the cytochrome CYP2E1 enzyme, and increase renal cellular damage in oUSCs. However, they did not affect mitochondrial mass, mtDNA, cellular senescence, or stem cell stemness. The effect of drug–drug interaction on chronic MtT^71^ requires further study and follow-up in clinical trials.

We performed a parallel comparison with conventional 2D monolayer cultures. It is important to note that USC could not be reliably maintained in a 2D environment for the full 6-week duration of our chronic exposure experiments due to significant cell detachment and loss. This instability underscores the necessity of the 3D scaffold for long-term toxicity studies.

Furthermore, our previous work has demonstrated the significant advantages of the USC-seeded silk scaffold model in maintaining physiological cell–cell and cell–matrix interactions, resulting in improved tissue-like morphology and functional marker expression over 2D systems.^11^^,^^15^ These established benefits support our use of the 3D scaffold for evaluating chronic drug toxicity. We acknowledge that a direct comparison of drug-induced effects between a stable 2D model (if developed) and our 3D system would provide compelling quantitative evidence for the necessity of this advanced platform. Future studies will be specifically designed to incorporate this direct comparison to explicitly quantify the protective or sensitizing effects of the 3D microenvironment on TFV toxicity.

In conclusion, our work demonstrates for the first time that TFV-triggered telomere-mitochondrial cascade leads to age-dependent renal progenitor cell impairment. In addition, PGC-1α is a critical regulator of mitochondrial function and integrity in renal progenitor cells from older adults on TFV formulation drugs. TFV-induced PGC-1α down-regulation contributes to telomere–mitochondrial dysfunction in renal progenitor cells from older adults. PGC-1α has the potential to become a novel predictor of early chronic renal insufficiency and a therapeutic target. PGC-1α activator may have therapeutic potential for telomere–mitochondrial dysfunction in the older population.

This study provides valuable insight into the chronic toxicity of TFV on human USC, but it is subject to several limitations that warrant consideration. First, while we assessed oxidative stress, apoptosis, and mitochondrial dynamics, we did not directly quantify mitophagy (*e.g.*, PINK1, Parkin, or flux-based assays), which would better define mitochondrial quality control under drug injury; future work will address this pathway. Second, we employed a supraphysiological TFV dose (∼10 × clinically relevant) to establish a safety margin and model worst-case scenarios (*e.g.*, impaired renal clearance, drug–drug interactions); this exposure exceeds typical patient steady-state levels and should be interpreted as an accelerated toxicology tool for defining the safety window rather than routine clinical conditions. Third, although we performed a parallel 2D monolayer arm, USCs could not be reliably maintained in 2D for the full 6-week exposure due to detachment and cell loss, underscoring the necessity of the 3D silk scaffold for long-term studies; prior work shows that USC-seeded silk scaffolds better preserve physiologic cell–cell and cell–matrix interactions, yielding more tissue-like morphology and functional marker expression than 2D systems. A head-to-head analysis against a stable 2D model (if developed) will be incorporated in future studies to quantify any protective or sensitizing effects of the 3D microenvironment on TFV toxicity. Finally, Western blotting was performed on scaffold-free USC spheroids (Micro-Tissue 3D Petri Dish) rather than on cells within the silk matrix to overcome inconsistent protein recovery from the dense, non-degradable scaffold; because cell–matrix context can influence expression, these blots should be viewed as qualitative mechanistic support for the primary functional and histological findings from the silk-scaffold model, and future work will optimize on-scaffold extraction or use complementary immunohistochemistry/immunofluorescence quantification.

## CRediT authorship contribution statement

**Pengfei Yu:** Writing – review & editing, Writing – original draft, Software, Resources, Methodology, Formal analysis, Data curation. **Huifen Ding:** Writing – review & editing, Software, Methodology. **Jian-Xing Ma:** Writing – review & editing, Formal analysis, Conceptualization. **Zhongping Duan:** Writing – review & editing, Supervision, Investigation, Conceptualization. **Yu Chen:** Writing – review & editing, Supervision. **Anthony Atala:** Writing – review & editing, Supervision. **Yuanyuan Zhang:** Writing – review & editing, Supervision, Resources, Funding acquisition, Data curation, Conceptualization.

## Ethics declaration

The study was conducted in accordance with the Declaration of Helsinki and approved by the Institutional Review Board (or Ethics Committee) of Wake Forest University School of Medicine (IRB Number: IRB00014033; approved on 09 March 2023).

## Data availability

All data that support the findings of this study are available in the paper and its supplemental information. Other raw data can be accessed upon request.

## Funding

This work was supported by the National Institute of Allergy and Infectious Diseases, National Institutes of Health, under Contract No. Funding Sources: NIAID, R21 AI152832, R03 AI165170; NEI, R21 EY0358332024; 2024 Translational Team Science Pilot Award, Translational Eye and Vision Research Center (TrEVR), Wake Forest School of Medicine (WFSOM); 2024 Pilot Research Grant, Eye Bank Association of America (EBAA); (PI: Y.Z).

## Conflict of interests

Yuanyuan Zhang is a member of *Genes & Diseases* Editorial Board. To minimize bias, he was excluded from all editorial decision-making related to the acceptance of this article for publication. The remaining authors declare no conflict of interests.
