# Supplementary material for: Age-dependent mitochondrial toxicity in renal stem cells: A precise 3D silk matrix model
Source: Genes Dis. 2026 Feb 23;13(6):102108. doi: 10.1016/j.gendis.2026.102108 (PMC13486992; doi:10.1016/j.gendis.2026.102108)
Supplement: Supplementary file 1 [file mmc1.docx]

**Figure S1.** In vitro cell cultures on 3D-SFM at two time points. (A) Scanning electron microscopy (SEM) examination of cells seeded on SFM. (B) yUSC and oUSC observed with an optical microscope at 3 days and 6 weeks. (C) Live/dead staining of yUSC, oUSC, HEK293, and HepG2 cells over the course of a 6-week 3D culture. (D-E) The cell numbers of yUSC, oUSC, HEK293, and HepG2 in 3D culture were assessed using the CCK-8 assay. (n = 6) Data are expressed as mean ± SD. Student's t-test was used. *p < 0.05; **p < 0.01. * versus 0.1% DMSO control in yUSC; ^#^ versus 0.1% DMSO control in oUSC.

| **Table S1.** Primers used in RT-PCR analysis. | | |
| --- | --- | --- |
| P21 | Forward | *TGTCCGTCAGAACCCATGC* |
|  | Reverse | *AAAGTCGAAGTTCCATCGCTC* |
| P53 | Forward | *TGTGACTTGCACGTACTCCC* |
|  | Reverse | *ACCATCGCTATCTGAGCAGC* |
| Rb | Forward | *TTGGATCACAGCGATACAAACTT* |
|  | Reverse | *AGCGCACGCCAATAAAGACAT* |
| MtDNA | Forward | *CACCCAAGAACAGGGTTTGT* |
|  | Reverse | *TGGCCATGGGTATGTTGTTA* |
| NuDNA | Forward | *TGCTGTCTCCATGTTTGATGTATCT* |
|  | Reverse | *TCTCTGCTCCCCACCTCTAAGT* |
| GAPDH | Forward | *CATGTTCGTCATGGGGTGAACCA* |
|  | Reverse | *AGTGATGGCATGGACTGTGGTCAT* |

| **Table S2.** Reagents used for Western blot analysis. | | | |
| --- | --- | --- | --- |
| Antibodies | Vendor | Dilution |  |
| Total OXPHOS (I-V) | Abcam, USA | 1:250 | ab110413 |
| SOD2 | Cell Signaling Technology, USA | 1:1000 | 13141 |
| KIM-1 | Abcam, USA | 1:1000 | ab47635 |
| CYP2E1 | Abcam, USA | 1:1000 | ab28146 |
| Caspase-3 | Abcam, USA | 1:1000 | 9662s |
| Cleaved Caspase-3 | Abcam, USA | 1:1000 | 9661s |
| PGC1-α | Santa Cruz, USA | 1:1000 | sc-517380 |
| TRF1 | Abcam, USA | 1:1000 | ab10579 |
| mtTFA | Santa Cruz, USA | 1:1000 | sc-166965 |
| Nrf1 | Santa Cruz, USA | 1:1000 | sc-28379 |
| Nrf2 | Santa Cruz, USA | 1:1000 | sc-365949 |
| β-Actin | Santa Cruz biotechnology | 1:1000 | sc-47778 |
| Goat anti-Rabbit | Abcam, USA | 1:5000 | ab6721 |
| Rabbit anti-Mouse | Abcam, USA | 1:5000 | ab6728 |
| **Abbreviations:** OXPHOS: Oxidative phosphorylation; SOD2: Superoxide dismutase 2; KIM-1: Kidney injury molecule-1; CYP2E1: Cytochrome P450 2E1; PGC1-α: Peroxisome proliferator-activated receptor gamma coactivator 1-alpha; TRF1: Telomeric repeat-binding factor 1; mtTFA: Mitochondrial transcription factor A; Nrf1: Nuclear respiratory factor 1; Nrf2: Nuclear respiratory factor 2 | | | |

Materials and methods:

1.1 Preparation of silk fiber matrix:

Reagents: silk cocoons; sodium carbonate (Na₂CO₃, 0.05 M); lithium bromide (LiBr, 9.8 M); distilled/DI water; 1,1,1,3,3,3-hexafluoro-2-propanol (HFIP); trifluoroacetic acid (TFA, used at 10% v/v in HFIP as indicated); absolute ethanol (EtOH, 100%); ammonium hydroxide (NH₃·H₂O); PBS.
Equipment. Hotplate/stirrer; dialysis tubing (MWCO ~3.5 kDa); −80 °C freezer; lyophilizer; electrospinning unit with high-voltage supply; syringe pump; blunt steel needle (yellow gauge); ethanol bath for wet collection; biopsy punches (4 mm, 8 mm).

1) Degumming (sericin removal).
   Cut 40 g cocoons into ~1–2 cm pieces. Boil twice in 1 L 0.05 M Na₂CO₃ at 100 °C for 30 min each; rinse thoroughly with DI water after each boil to neutrality. Air-dry the degummed silk fibers.
2) Fibroin dissolution.
   From the dried fibers, dissolve 20 g silk in 100 mL 9.8 M LiBr at 60 °C for 1 h, then maintain 60 °C/350 rpm on a magnetic hotplate stirrer for an additional 3 h until homogeneous.
3) Dialysis (LiBr removal).
   Transfer the SF/LiBr solution into MWCO ~3.5 kDa dialysis tubing and dialyze against distilled water at 4 °C: change water 3× on day 1, then 2×/day for 3 additional days.
4) Freeze and lyophilize to SF sponge.
   Aliquot the dialyzed SF solution into 50 mL tubes and freeze at −80 °C. Remove caps and lyophilize for 7 days to obtain silk fibroin sponges.
5) Spinning solution (dope) preparation.
   Dissolve 1.5 g SF sponge in 10 mL HFIP containing 10% v/v TFA (HFIP:TFA = 90:10) until optically homogeneous (room temperature, several hours). Load into a 5 mL syringe fitted with a yellow-gauge blunt needle.
   Note: This corresponds to ~15% (w/v) SF. Where a 10% (w/v) dope was used, we prepared 1.0 g SF in 10 mL HFIP ± TFA with identical downstream parameters.
6) Electrospinning and wet collection.
   Electrospin with flow rate 0.5 mL/h, voltage 20 kV, tip-to-collector distance 10 cm (ambient 25–30 °C, 30–40% RH). Collect fibers directly into 100% ethanol (wet collection) to form random mats; aligned mats can be obtained using a partially submerged rotating mandrel as needed.
7) Crosslinking and post-processing.
   Keep the wet-collected mats in 100% EtOH for ≤ 45 min to induce β-sheet formation. Rinse in fresh ethanol, then with NH₃·H₂O/ethanol (optional) and multiple DI-water washes. Transfer to DI water, freeze at −80 °C, and lyophilize for 3 days to obtain porous 3D SFM.
8) Formatting and sterilization.
   Punch to target sizes: s-SFM: 4 mm × 0.2 mm (96-well assays); l-SFM: 8 mm × 1 mm (WB/qPCR). Sterilize by 70% ethanol soak and UV exposure, followed by sterile PBS rinses.

1.2 Protein Analysis Rationale:

The methods section specifies that protein analysis by Western blot was performed using the Micro-Tissue 3D Petri Dish system to generate cellular lysates, and we explicitly confirm that the silk scaffold was not used in these specific assays because lysing the cells grown directly on the dense, non-degradable silk matrix for quantitative protein recovery proved challenging and resulted in highly inconsistent yields; instead, protein quantification was performed on scaffold-free spheroids generated by the Micro-Tissue system, with the primary goal of this assay being to compare the mitochondrial function markers between drug-treated USC-TM and the untreated control USC-TM. We acknowledge the potential for differences in protein expression between cells on the silk matrix and the scaffold-free spheroids due to variations in cell-matrix interactions; however, the Western blot analysis was intended to provide mechanistic, qualitative confirmation of the drug's effect on key signaling pathways (e.g., apoptosis, oxidative stress, mitochondrial function) identified by the functional and histological data obtained from the silk-scaffold model, rather than quantifying absolute protein levels, and thus the observed changes in pathway markers are considered relevant for substantiating the main findings.
